# Serial T-SPOT.TB responses in Tanzanian adolescents: Transient, persistent and irregular conversions

**DOI:** 10.1371/journal.pone.0268685

**Published:** 2022-06-24

**Authors:** Maryam A. Amour, Christiaan A. Rees, Patricia J. Munseri, Jamila Said, Albert K. Magohe, Mecky Matee, Elizabeth A. Talbot, Robert D. Arbeit, Kisali Pallangyo, C. Fordham von Reyn

**Affiliations:** 1 Muhimbili University of Health and Allied Sciences, Dar es Salaam, Tanzania; 2 Geisel School of Medicine at Dartmouth, Hanover, New Hampshire, United States of America; 3 Department of Medicine, Brigham and Women’s Hospital, Boston, Massachusetts, United States of America; 4 Tufts University School of Medicine, Boston, Massachusetts, United States of America; McGill University, CANADA

## Abstract

**Background:**

Prospective studies of interferon-gamma release assays (IGRA) on healthy subjects in tuberculosis-endemic regions have not examined the long-term variability of serial assays. This issue is relevant to the interpretation of tuberculosis (TB) vaccine trials based on prevention of infection.

**Methods:**

T-SPOT.TB assays were performed manually on healthy adolescents during a tuberculosis vaccine trial in Tanzania at 5 intervals over 3 years. Assay results were defined as negative, positive, borderline or invalid. Subsequently, microtiter plates were analyzed by an automated reader to obtain quantitative counts of spot forming cells (SFCs) for the present analysis.

**Results:**

3387 T-SPOT.TB samples were analyzed from 928 adolescents; manual and automated assay results were 97% concordant. Based on the quantitative results 143 (15%) participants were prevalent IGRA-positives at baseline, were ineligible for further study. Among the remaining IGRA-negative participants, the annual rate of IGRA conversion was 2·9%. Among 43 IGRA converters with repeat assays 12 (28%) were persistent converters, 16 (37%) were transient converters, and 15 (35%) comprised a new category defined as irregular converters (≥2 different subsequent results). ESAT-6 and CFP-10 responses were higher in prevalent than incident positives: 53 vs 36 for CFP-10 (*p* < 0·007); 44 vs 34 for ESAT-6 (*p* = 0·12).

**Conclusions:**

Definitions of IGRA conversion, reversion, and persistence depend critically on the frequency of testing. Multiple shifts in categories among adolescents in a TB-endemic country may represent multiple infections, variable host responses in subclinical infection, or assay variation. These findings should to be considered in the design and interpretation of TB vaccine trials based on prevention of infection. Household contact studies could determine whether even transient IGRA conversion might represent exposure to an active case of *M*. *tuberculosis* disease.

## Introduction

The World Health Organization (WHO) has recently targeted identification and treatment of persons with latent tuberculosis infection (LTBI) as a critical component of TB control in countries with both low and high TB incidence [1]. LTBI is diagnosed by the presence an immune response to mycobacterial antigens using the tuberculin skin test (TST) or one of two interferon-gamma (IFN-γ) release assays (IGRA): the T-SPOT.TB assay and the QuantiFERON-TB Gold Plus [2–4]. Prospective studies have raised questions about the significance of prevalent vs incident infection as defined by an IGRA as well as about the significance of reversion from IGRA-positive to IGRA-negative [5–8]. Most studies from TB-endemic regions have analyzed only the results of one baseline and one repeat IGRA assay and thus have not addressed these issues.

In a recently completed trial of the investigational TB vaccine DAR-901, we performed ≥ 5 serial T-SPOT.TB assays over three years on a cohort of adolescents in Tanzania, and used manual counts of Spot-Forming Cells (SFCs, manual limit 20 SFCs) to define study endpoints [9]. Subsequently, SFCs were quantified by an automated reader capable of enumerating >20 SFCs. We analyzed quantitative SFC responses to the four T spot TB antigens and identified frequent shifts in IGRA categories over time including a new pattern of multiple shifts that we define as irregular conversion. Our findings have potential implications for Prevention of Infection (POI) trials of new TB vaccines and for the use of IGRA conversion in tuberculosis case-finding.

## Methods

### Study population and trial procedures

BCG-immunized adolescent volunteers aged 13–15 years were recruited from secondary schools in Dar es Salaam between April and October 2016. The trial was approved by the Research Ethics Committee at Muhimbili University of Health and Allied Sciences. Written informed consent was obtained from all parents or guardians and written informed assent was obtained from all adolescent volunteers. Participants who were T-SPOT.TB IGRA-positive at baseline were ineligible for immunization, further follow-up, or repeat IGRAs. IGRA-negative adolescents were enrolled in the immunization phase of the trial between April 2016 and March 2017.

Injections of vaccine or placebo were administered at zero, two and four months. IGRA testing for all subjects was performed at: baseline (screening), two months, and one, two, and three years with follow-up completed by December 2019. Participants who converted to IGRA-positive were scheduled for an additional repeat IGRA three months later. Since immunization did not affect the primary endpoint of new TB infection (IGRA conversion) or the secondary endpoint of persistent new TB infection (persistent IGRA conversion) we have pooled results from vaccine and placebo recipients [9]. The trial was not designed to evaluate the long term risk of progression to TB disease after IGRA conversion.

### IGRA interpretation and classification of subjects

Assay results were categorized as positive, borderline, negative, or invalid based on the manufacturer’s criteria (Oxford Immunotec, Oxford, UK) (**Table 1)**. In a systematic review the sensitivity and specificity of automated T-SPOT.TB readings were found to be 90% and 93% respectively [10]. Each T-SPOT.TB assay includes four wells: a negative control (Nil) consisting of sterile media, a positive control (PHA) consisting of phytohemagglutinin, and two wells consisting of TB early secreted antigen-6 (ESAT-6) and TB culture filtrate protein (CFP-10). Two laboratory technicians in Tanzania were trained by Oxford Immunotec before the start of the study and again after one year. Spot forming counts (SFCs) were determined manually in Tanzania using a microscope (manual IGRA, mIGRA), and these results defined the per-protocol study endpoints reported for the trial [9]. For the per- protocol manual interpretation reported in the trial, the technician was instructed to count SFCs only up to 20 per well and categorize all other wells as “>20.” Microtiter plates were subsequently sent to Oxford Immunotec (Oxford, UK) for quantitative reading using an automated plate reader with the capability to count >20 SFCs up to the resolution capacity of the instrument (quantitative IGRA, qIGRA). The present report is based on analysis of these quantitative responses which are considered the gold standard for T-SPOT.TB interpretation.

**Table 1:** Interpretation of T-SPOT TB results based on spot-forming cells (SFCs).

Interpretation	Nil	ESAT-6 or CFP-10 minus Nil	PHA
	(Negative Control)		(Positive Control)
Positive	≤ 10 spots	> 7 spots	Any number
Borderline	≤ 10 spots	5 to 7 spots	Any number
Negative	≤ 10 spots	< 5 spots	≥ 20 spots
Invalid	≤ 10 spots	< 5 spots	< 20 spots
	> 10 spots	Any number	Any number

ESAT-6: TB early secreted antigen-6; CFP-10: TB culture filtrate protein-10; PHA: phytohemagglutinin. Values represent counts of Spot-forming Cells (SFCs).

**Fig 1:**
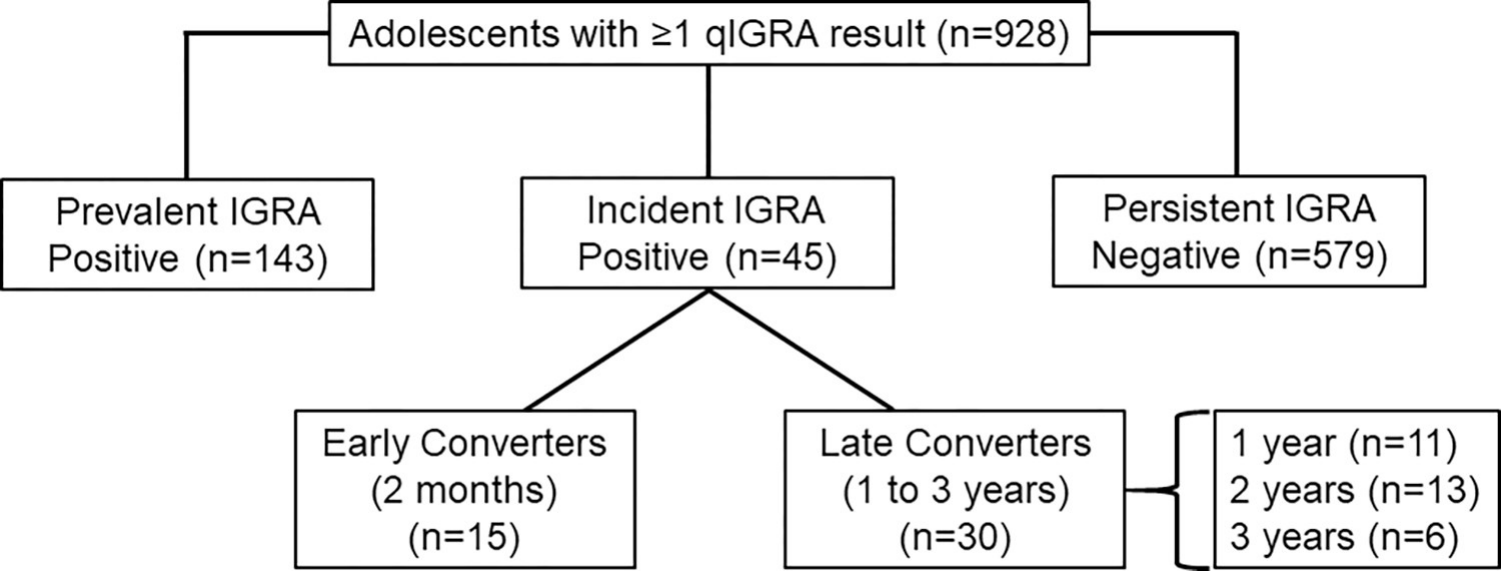
Analysis groups based on quantitative IGRA results. Among 928 subjects with ≥1 qIGRA result, 161 (17.3%) failed to meet strict criteria for inclusion in an analysis group, including 107 (11.5%) who were negative at baseline, but did not participate in the vaccine study and had no subsequent samples, 23 (2.5%) who had borderline or invalid baseline qIGRA, 19 (2.0%) whose only post-baseline non-negative qIGRA was borderline, and 12 (1.3%) who were excluded from the vaccine study because their baseline mIGRA was borderline or positive, but their qIGRA was not positive.

Prevalent IGRA positives were defined as subjects who were IGRA-positive at the time of baseline screening; these subjects were excluded from the vaccine trial and further follow-up. Subjects who were IGRA-negative at screening and converted to IGRA-positive during the three-year follow-up period were defined as IGRA converters. IGRA converters were further sub-divided into early converters (initial positive at two months) or late converters (initial positive at one, two, or three years). Based on IGRA results after the initial positive, subjects were further classified as transient converters (following initial positive, all subsequent IGRA results were negative), persistent converters (following initial positive, all subsequent IGRA results were positive), or irregular converters (following initial positive, subsequent IGRA results included ≥2 different results). Individuals without a repeat IGRA after their initial positive were excluded from that analysis. Persistent negatives were defined as participants who remained IGRA-negative throughout the entire follow-up period (**Fig 1)**.

IGRA-positive participants were evaluated for symptoms of TB disease using the Tanzanian TB Screening Questionnaire (TSQ) [11], and participants with positive TSQ responses were referred to National TB and Leprosy Program (NTLP) clinics for evaluation and treatment.

### Statistical analyses

All statistical analyses were performed using R version 3·5·0. Fisher’s exact test was used for the comparison of categorical variables and the Mann-Whitney U-test was used for the comparison of count variables (*e*.*g*., the number of SFCs). For continuous variables, the Shapiro-Wilk test was used evaluate whether the data was consistent with a normal distribution. For apparently normally distributed data, the parametric Student’s *t*-test (for the comparison of two groups) and/or ANOVA (for the comparison of three or more groups) were used, while for non-normally distributed data, the non-parametric Mann-Whitney U-test (two groups) and/or Kruskal-Wallis test (three or more groups) were used. A *p*-value of 0.05 was considered the threshold for statistical significance. Cohen’s Kappa was used to evaluate the concordance between manual and automated IGRA interpretations. Since immunization did not affect rates of IGRA conversion both vaccine and placebo recipients were aggregated for the present analysis [9].

## Results

### Concordance between manual and quantitative T-SPOT TB readings

In total, 3387 T-SPOT TB samples with paired manual (mIGRA) and quantitative (qIGRA) results were analyzed in 928 individuals. Overall, the two methods were concordant for 3287 (97%) of the samples (Cohen’s Kappa = 0·844, 95% CI: 0·815–0·873) (**Table 2**). Among the 100 samples with different results by the two methods, 68 represented shifts from positive or negative manual readings to borderline or invalid automated readings. A change from negative to positive readings was seen only for two (2) samples and from positive to negative only for nine (9) samples. These changes in IGRA categories did not impact conclusions about vaccine efficacy [9]. The present analysis of IGRA converter categorizations **(Fig 1)** is based exclusively on all available quantitative IGRA results.

**Table 2:** Concordance between results obtained by quantitative IGRA and manual IGRA.

		N	Quantitative IGRA			
			Positive	Borderline	Negative	Invalid
			(N = 254)	(N = 59)	(N = 3025)	(N = 49)
			n (%)	n (%)	n (%)	n (%)
Manual IGRA	Positive	**278**	**251** (99%)	**14** (24%)	**9** (<0.1%)	**4** (8%)
	Borderline	**38**	**1** (<0.1%)	**22** (37%)	**15** (<0.1%)	**0** (0%)
	Negative	**3049**	**2** (<0.1%)	**22** (37%)	**2997** (99%)	**28** (57%)
	Invalid	**22**	**0** (0%)	**1** (2%)	**4** (<0.1%)	**17** (35%)

mIGRA: Manual IGRA interpretation; qIGRA: Quantitative IGRA interpretation using an automated plate reader. A total of 3,387 assays were analyzed by both methods. Light grey cells correspond to concordance between qIGRA and mIGRA results.

### Baseline characteristics of participants

Among 928 adolescents, 767 were classified by qIGRA as either: prevalent IGRA positives (n = 143), IGRA converters (n = 45), or persistent IGRA negatives (n = 579) (**Fig 1**). Baseline characteristics of study participants in the three groups of interest are presented in **Table 3**. There were no significant between-group differences with respect to sex, BMI, white blood cell (WBC) count, platelet count, school district, or the number of spot-forming counts in the Nil or PHA wells. Baseline hemoglobin was significantly lower in IGRA converters than either prevalent positives or persistent negatives.

**Table 3:** Baseline characteristics of study participants.

	Prevalent IGRA Positives (n = 143)	Incident IGRA Converters (n = 45)	Persistent IGRA Negatives (n = 579)	*p*-value
Mean Age [a] (Years [SD])	14·3 [0·75]	13·9 [0·78]	14·2 [0·75]	0·23
Sex (n (%))[b]				0·28
Male	69 (48·3)	16 (35·6)	248 (42·8)	
Female	74 (51·7)	29 (64·4)	331 (57·2)	
BMI [c]	19·1	18·5	19·3	0·52
(Median [Range])	[13·8–34·9]	[12·9–33·1]	[13·3–35·4]	
Hemoglobin [c]	12·7	11·9	12·6	<0·001
(Median [Range])	[8·7–17·1]*	[10·0–20·9]*^,^^†^	[8·6–23·5]^†^	
WBC count [c]	6·0	5·4	5·8	0·54
(Median [Range])	[3·3–12·0]	[2·6–9·7]	[2·8–16·5]	
Platelet count [c]	304	312	300	0·75
(Median [Range])	[125–672]	[115–719]	[88–1914]	
District (n (%)) [b]				0·73
Temeke	101 (70·6)	35 (77·8)	428 (73·9)	
Ilala	5 (3·5)	1 (2·2)	24 (4·2)	
Kigamboni	4 (2·8)	1 (2·2)	25 (4·4)	
Ubungo	33 (23·1)	8 (17·8)	102 (17·6)	
Baseline qIGRA SFCs [c]				
(Median [Range])				
Nil	0 [0–6]	0 [0–4]	0 [0–6]	0·27
ESAT-6	24 [0–322]*^,^^†^	0 [0–7]*	0 [0–4]^†^	<0·001
CFP-10	28 [0–438]*^,^^†^	0 [0–5]*^,^^ǂ^	0 [0–7]^†^^,^^ǂ^	<0·001
PHA	228 [21–521]	203 [22–530]	206 [20–586]	0·21

p-values reflect the comparison across all three groups.

*,†, and ǂ all indicate pairwise comparisons with *p*-values <0.05.

[a] ANOVA

[b] Chi-squared test

[c] Kruskal-Wallis test

The annual rate of qIGRA conversion from negative to positive (i.e., TB infection) was 2·9 (95% CI: 2·2–3·9) per 100 person-years and was not associated with significant differences in baseline characteristics. The rate of qIGRA conversion among subjects with normal hemoglobin (≥12 g/dL) at baseline was 1·9 (95% CI: 1·3–3·0), significantly lower than for those with mild (11·9 to 11·0 g/dL) or moderate (10·9 to 8·0 g/dL) anemia (4·3 [2·6–7·2] and 11·2 [5·6–22·4], respectively; *p* < 0·0001).

### Characteristics of incident IGRA converter subgroups

The 45 subjects who converted from IGRA-negative to IGRA-positive included 15 who converted prior to receiving the second vaccine dose at 2 months (early converters), and 30 who converted at or following the one-year follow-up visit, scheduled for eight months after completion of the three-dose vaccine series (late converters). There were no differences between these groups with regard to baseline characteristics.

Analysis of the SFCs for the four assay wells at baseline and at the first positive assay is shown in **Table 4**. There were no significant differences between early and late converters at either timepoint. At baseline, CFP-10 SFCs among the early converters were significantly greater than among those who remained persistent negatives (1·6 vs 0·5, *p* < 0·001).

**Table 4:** Characteristics of IGRA converters based on timing of initial positivity.

(N = 45)	Early Converters	Late Converters	*p*-value
	(n = 15)	(n = 30)	
Age in Years	15 [13–15]	14 [13–15]	**0**·**001**
(Median [Range])			
Baseline qIGRA SFCs			
(Median [Range])			
Nil	0 [0–3]	0 [0–4]	0·43
ESAT-6	0 [0–5]	0 [0–7]	0·41
CFP-10	1 [0–4]	0 [0–5]	0·08
PHA	195 [40–530]	204 [22–527]	1·00
Incident qIGRA SFCs			
(Median [Range])			
Nil	0 [0–5]	0 [0–2]	0·15
ESAT-6	12 [2–133]	14 [1–277]	0·65
CFP-10	10 [0–405]	20 [0–224]	0·15
PHA	330 [75–542]	397 [112–1000]	0·12

Analysis of Nil, ESAT-6, and CFP-10 was done using the Mann-Whitney U-test; analysis of PHA was done using the Student’s t-test. Early converters: initial positive at month two. Late converters: initial positive at one to three years.

Among the 45 IGRA converters, 43 (96%) had at least one subsequent IGRA following their initial positive result (2 converters were lost to follow-up). Of these, 16 (37%) were consistently IGRA-negative following their initial positive (transient converters), 12 (28%) were consistently IGRA-positive (persistent converters), and 15 (35%) had ≥2 different assay results after IGRA conversion (irregular converters) **(S1 Table).** Thus, irregular converters could not be defined as either persistent reverters (transient positives) or persistent positives. There were no significant differences between transient, persistent, and irregular converters with respect to any baseline characteristics. Analysis of the SFCs for the four assay wells at baseline and at the first positive assay is shown in **Table 5**.

**Table 5:** IGRA results at baseline and at time of first positive IGRA for subgroups of converters.

	Transient Converters	Persistent Converters	Irregular Converters	*p*-value
	(n = 16)	(n = 12)	(n = 15)	
Baseline qIGRA SFCs				
(Median [Range])				
Nil	0 [0–3]	0 [0–2]	0 [0–4]	0·61
ESAT-6	0 [0–5]	0 [0–2]	1 [0–7]	0·10
CFP-10	0 [0–4]	0.5 [0–4]	1 [0–5]	0·06
PHA	198 [30–496]	220 [32–527]	195 [22–530]	0·92
Incident qIGRA SFCs				
(Median [Range])				
Nil	0 [0–2]	0 [0–5]	0 [0–3]	0·69
ESAT-6	11.5 [2–277] ^ǂ^	40 [4–133] ^ǂ^	12 [2–48]	0·17
CFP-10	10 [0–224]*	25 [6–405]*	12 [0–58]	0·09
PHA	323 [81–578]	385 [118–1000]	330 [75–634]	0·70

Converter subcategories are based on the pattern of IGRA results obtained after the initial positive result: Transient converters were subsequently consistently IGRA-negative; persistent converters were consistently IGRA-positive; irregular converters had ≥2 different assay results after conversion.

Results in the *p*-value column represent analyses across all three groups using Kruskal-Wallis test, except for the baseline PHA analysis, which was done by ANOVA. Comparison of transient vs persistent converter subgroups was performed using the Mann-Whitney U-test, except for the baseline PHA analysis, which was done by Student’s t-test, with results shown by symbol

^ǂ^*p* = 0.25

*p = 0.06. See text regarding potential effect of vaccine on ESAT-6.

The proportion of converters meeting a definition of irregular was related to the number of repeat IGRAs performed: 12 (63%) of 19 with ≥3 repeat IGRAs vs 3 (20%) of 15 with 2 repeat IGRAs (p <·001, **Table 6**). Among the 15 irregular converters six progressed from IGRA conversion to IGRA reversion to a second IGRA conversion (**Table 7**). SFC counts for the 41 incident converters at the 5 per-protocol visits are shown in **S2 Table.**

**Table 6:** Distribution of 3 IGRA conversion categories based on number of IGRAs performed after initial conversion.

N of IGRAs after initial conversion	Transient Converters (n = 16)	Persistent Converters (n = 12)	Irregular Converters (n = 15)	Total
1	5	4	0	9
2	9	3	3	15
3	0	1	5	6
4	2	3	1	6
5	0	0	4	4
6	0	1	2	3
Total	16	12	15	43

Excludes two initial converters with no repeat IGRA assays.

*P* = 0.004 for distribution by Chi-squared analysis.

**Table 7:** Results of serial IGRAs performed among 15 subjects who met criteria for irregular converters.

Subject		Visit								
	Baseline	2 Months	A	B	1 Year	C	2 Years	D	36	E
1	N	N	-	-	P	P	P	-	B	-
2	N	N	-	-	N	-	P	P	P	N
3	N	P	N	B	N	-	N	-	N	-
4	N	P	N	N	N	-	P	N	-	-
5	N	P	N	N	B	-	-	-	-	-
6	N	P	N	-	B	-	N	-	P	N
7	N	N	-	-	N	-	P	N	P	-
8	N	N	-	-	N	-	P	P	N	-
9	N	P	N	-	N	-	P	N	-	-
10	N	P	-	-	B	-	N	-	N	-
11	N	N	-	-	P	-	P	P	N	-
12	N	P	P	N	N	-	N	-	B	N
13	N	P	N	B	P	-	B	-	-	N
14	N	-	-	-	P	-	B	-	P	-
15	N	P	P	N	N	-	P	N	N	-

Irregular converters were defined as subjects who converted from IGRA-negative to IGRA positive then had ≥2 subsequent shifts in IGRA category. Per-protocol visits were scheduled at baseline, 2 mos and 1, 2 and 3 years. Additional visits between these times are labelled A, B, C, D and E.

B = Borderline

N = Negative

P = Positive

### Characteristics of IGRA results in incident positives and prevalent positives

**Fig 2:**
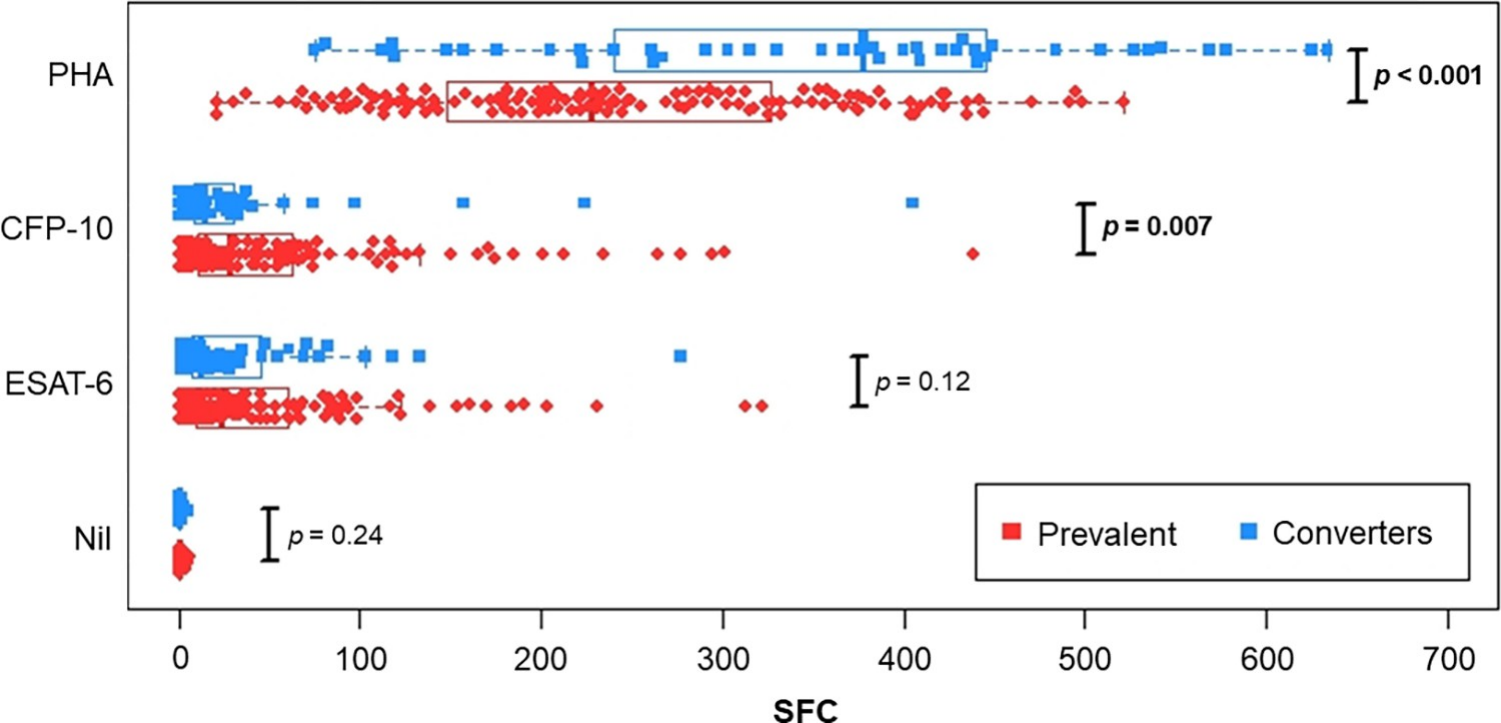
Distribution of SPCs comparing prevalent positives and first positive in converters. Prevalent positives (*n* = 143, red diamonds) and IGRA converters (*n* = 45, blue squares) at time of first positive IGRA (including both early and late converters as defined in the text). For converters with more than one positive qIGRA, only the results of the first positive qIGRA are included. *p*-values are calculated using the Mann-Whitney U-test. See text for details of potential impact of vaccine on the ESAT-6 results.

Prevalent positives had significantly more SFCs in the CFP-10 well than IGRA converters (53·1 vs. 35·9, *p* = 0·007) and a trend toward more SFCs in the ESAT-6 well (44·0 vs. 34·5, *p* = 0·12) (**Fig 2**). The difference in responses to the two antigens may reflect the previously reported observation that IGRA converters who received the DAR-901 vaccine had higher ESAT-6 SFCs than IGRA converters who received placebo [9]. IGRA converters had significantly more SFCs in the PHA well than prevalent positives (365.1 vs 240·9, *p* < 0·001). This difference may reflect the broad inflammatory and immune stimulation accompanying recent infection with TB that has been contained without progression to acute disease.

## Discussion

In this three-year prospective study, using conversion of the T-SPOT.TB IGRA as a marker of new TB infection, we identified prevalent infection in 15% of participants and an annual rate of IGRA conversion of 2·9%. In contrast to most other longitudinal IGRA studies, participants had a minimum of five separate IGRA assays over three years; sequential IGRA results were remarkably dynamic. Over 60% of converters reverted to negative and almost half of the reverters had one or more subsequent positive and/or borderline result. Further, SFC responses to TB antigens were higher among prevalent than incident positives. Taken together these observations raise challenging questions about the appropriate interpretation of a positive IGRA in different contexts.

Latent TB–defined as a prevalent positive TST in an asymptomatic, healthy individual–is, in most persons, associated with protection against TB disease. Studies of Norwegian nurses in the pre-IGRA era showed that those who began work at a TB hospital with a positive (prevalent) TST had a lower risk of acquiring new TB disease than nurses with a negative TST; new TST converters to positive had the highest risk to TB disease [12]. In aggregate, numerous studies indicate that a baseline positive TST is associated with approximately 79% protection against TB disease from subsequent exposure [13]. We hypothesize that a prevalent positive IGRA may be associated with analogous protection.

In contrast, an incident positive TST (documented conversion from negative) is associated with a substantially increased risk of progression to active TB over the next two to five years [5] (18). After that period, the positive TST persists, effectively becoming a prevalent positive.

A single (prevalent) positive IGRA identifies subjects at an increased risk of progressing to active TB compared to those with a negative IGRA. But the predictive value is <5%, indicating most subjects with a prevalent IGRA are protected [14–17]. Applying higher thresholds to define a positive test increases the predictive value substantially, but not to a level of major clinical utility [7, 15]. Data on the risk of subsequent TB disease in baseline prevalent positives compared to new converters is available from 2 studies conducted in South Africa. In a trial among adolescents using the QuantiFERON-TB IGRA assay, prevalent positives showed a lower risk of progression to TB than incident positives (0·97/100 person-years vs. 1·39/100 person-years) [7]. Unlike the T-spot TB assay, the QuantiFERON-TB assay used in the South African studies does not have a borderline category, an issue that was addressed in analysis of data from 5 South African cohorts [18]. Whereas the manufacturer specifies a single, discrete value to define a negative vs. positive assay, the study proposed an "uncertainty zone" around that value (in effect, a borderline category) to differentiate results more sharply. The stricter criteria were used to define "stringent" populations of non-converters, persistent (prevalent) positives, and converters [18]. Thus defined, those populations had greater concordance with TST results and significantly differentiated risks of progression to TB disease. As in the other study from South Africa the rate of TB was lower for stringent persistent positives, 0.97 (p = 0.005) than for stringent converters 1.60 (p = 0.0003). The study concluded that results in the uncertainty zone potentially represented both "immunological and assay variability" [18].

It is not clear if the risk of TB following an incident positive depends on whether the conversion is transient (reversion) or persistent. In the two studies cited above using the QuantiFERON-TB assay (7, 19), the number of reverters was limited and the definitions differed. Overall, those studies noted that prevalent positives had stronger assay responses than incident positives, and that among converters weaker assay responses were more commonly associated with reversion. There were fewer cases of incident TB disease among the reverters, but with the limited numbers the difference was not statistically significant. In our study using the T-SPOT.TB assay, the magnitude of the SFC response for each TB Ag was also higher among prevalent positives compared to incident positives (converters) and higher among persistent converters than among transient or irregular converters.

Across all three reports referenced above, the results of serial IGRA tests showed considerable variability. In an additional study among health care workers in India results of 4 serial IGRA tests showed substantial variability that could not be related to differences in TB exposure [19]. In our study, in which initially IGRA-negative subjects had up to six (6) IGRAs following conversion to positive, repeated testing identified a new category of irregular conversion, defined by serial samples demonstrating multiple shifts among the categories of positive, negative, and/or borderline. As with simple reversion, both the contributing factors and the long-term clinical implications of this pattern are unknown. Of note, we observed six (6) subjects who reverted to negative with a subsequent positive assay one to three years later, a pattern consistent with multiple episodes of infection in a TB endemic country (S1 Data). Additional factors potentially contributing to both transient and irregular conversions include variability in the host immune response to subclinical infection and assay variability. Whatever the mechanisms, multiple studies now indicate that the number of assays, the sampling interval, and the definition of a positive assay can impact the detection of prevalent and incident positives, derived rates of infection, and the predictive value of those results for development of TB disease.

Overall, 15% of adolescents were prevalent positives and were excluded from the trial and further follow-up. Among eligible Tanzanian adolescents followed for up to 3 years the annual rate of incident positives was 2.9%. That rate is comparable to the 2.6% (95% CI 2.2–3.1) rate reported among adolescents in Kenya [20], but lower than the 4% to 14% rates reported in South Africa [7, 21]. With only a single IGRA-assay we cannot determine with certainty how many of the prevalent positives in our trial represent persistent positives. However, the 2.9% annual IGRA conversion rate suggests that a majority of the 15% of prevalent positives had been infected for more than 2 years, the period of greatest risk for progression to TB disease [22]. As shown for prevalent positives in other studies, we hypothesize that most of the prevalent IGRA positives in our study represent a favorable, stable, protective phenotype in a TB-endemic region with the potential for multiple episodes of re-exposure.

Household contact studies of adolescent TB disease have concluded that most disease is acquired outside the home [23]. However, TB disease may represent TB infection acquired two to five years earlier, by which time a household source case may no longer have active TB or even be present [22]. Serial IGRA testing to identify adolescents with IGRA conversion might provide a more timely marker of TB transmission in the household from an active case of TB disease. In addition, these studies could determine whether even transient IGRA conversion might represent exposure to a case of infectious TB disease in the household. Our experience shows that ongoing school-based IGRA testing of adolescents is feasible and might serve as an innovative case finding method to identify contacts with infectious TB in the household or elsewhere.

We found a high degree of concordance between manual and quantitative IGRA results with an overall agreement at 97% with a kappa coefficient of 0·84, similar to other studies, and important in low-income settings [24]. Serial testing to identify IGRA converters in TB endemic regions may provide a novel approach to identifying risk factors for infection [25]. We found that baseline hemoglobin levels were quantitatively lower in future IGRA converters suggesting that this is a biologically relevant effect. Iron deficiency anemia has been associated with impairment in humoral and cell-mediated immunity [26]

This study has several limitations. Long term follow-up to determine the future risk of TB was not part of the study design in a POI trial. Additionally, we were only able to determine quantitative SFCs routinely at annual intervals or 3 months after a new conversion, indicating that with the exception of those who converted 2 months after enrollment, the results observed for new conversions may have represented responses between 3–11 months later. Testing at more frequent time intervals (e.g., every 6 months) might have revealed even higher levels of variability. Adolescents with new conversion were tested more frequently than those without, a protocol feature which resulted in a greater likelihood of detecting reversion in new converters. Finally, HIV testing was not performed for healthy adolescents in this trial because they are a low risk group in Tanzania and discussion with our community advisory group indicated that requiring testing could severely compromise recruitment [9].

## Conclusions

In conclusion, this three-year longitudinal study with five or more IGRA determinations per subject has shown that annual rates of IGRA conversion among adolescents in Dar es Salaam, Tanzania are significant at 2.9%. The majority of new converters subsequently reverted to negative or showed a newly defined irregular pattern with two or more different results subsequently. Thus, the definition of persistent IGRA positive was highly dependent on the frequency of sampling. Multiple shifts from negative to positive in the same subject were potentially consistent with multiple infections. Immune responses to the two IGRA TB antigens were strongest in prevalent positives who had not already progressed to active TB suggesting that those prevalent responses might be analogous to prevalent TST positives which have been shown to protect against TB disease on subsequent exposure. Serial IGRA testing of adolescents attending school was shown to be feasible and to have the potential for more timely detection of newly acquired TB infection. With the availability of point-of-care IGRA assays now in development identification of IGRA conversion in adolescents could be an innovative approach to detecting undiagnosed source TB disease cases in their household and community contacts.

## Supporting information

S1 Data (XLSX)

S1 Table Patterns of negative, borderline, positive, and indeterminant quantitative IGRA (qIGRA) results among study subjects at 5 per-protocol visits. IGRA result: negative = green; borderline = orange; positive = red; indeterminant = grey. White cells indicate the number of subjects who were lost to follow-up and are cumulative over time, but exclude subjects who missed an appointment and returned for a subsequent visit. For clarity, the number of subjects listed as “lost to follow-up” at each time point are in reference to their *baseline* IGRA value (*e*.*g*., at three years, 273 of 761 baseline negative subjects were lost to follow-up). Unscheduled and repeat visits (*n* = 97) are omitted for clarity. “Lost to follow-up” counts are cumulative over time. ^a^ Includes one subject who was positive on unscheduled visit (after visit 6) and positive on final visit; ^b^ Includes one subject who was negative on unscheduled visit (after visit 6) and negative on final visit; ^c^ Includes one subject who was positive on unscheduled visit (after visit 6) and negative on final visit; ^e^ Includes one subject who was positive on unscheduled visit (between one-year and two-year visit).
(DOCX)

S2 Table Spot forming cells (SFCs) among 41 incident IGRA converters and 579 persistent IGRA negatives. Values are reported as: median [range]. Subjects with first positive IGRA at an unscheduled visit (*n* = 4) are excluded. SFCs for the Nil, ESAT-6, CFP-10, and PHA wells were compared between two-month (*n* = 15), one year (*n* = 10), two year (*n* = 13), and three year (*n* = 3) positives at the time of initial positivity (green boxes) using the Kruskal-Wallis H-test. Results were as follows: Nil: *p* = 0.56, ESAT-6: *p* = 0.89, CFP-10: *p* = 0.11, PHA: *p* = 0.01. In pairwise comparison, PHA was significantly greater in year one positives versus two-month positives (*p* = 0.003). None of the other pairwise comparisons were statistically significant.
(DOCX)
