# Supplementary material for: Serial T-SPOT.TB responses in Tanzanian adolescents: Transient, persistent and irregular conversions
Source: PLoS One. 2022 Jun 24;17(6):e0268685. doi: 10.1371/journal.pone.0268685 (PMC9231806; doi:10.1371/journal.pone.0268685)
Supplement: S1 Table — IGRA result: negative = green; borderline = orange; positive = red; indeterminant = grey. White cells indicate the number of subjects who were lost to follow-up and are cumulative over time, but exclude subjects who missed an appointment and returned for a subsequent visit. For clarity, the number of subjects listed as “lost to follow-up” at each time point are in reference to their baseline IGRA value (e.g., at three years, 273 of 761 baseline negative subjects were lost to follow-up). Unscheduled and repeat visits (n = 97) are omitted for clarity. “Lost to follow-up” counts are cumulative over time. a Includes one subject who was positive on unscheduled visit (after visit 6) and positive on final visit; b Includes one subject who was negative on unscheduled visit (after visit 6) and negative on final visit; c Includes one subject who was positive on unscheduled visit (after visit 6) and negative on final visit; e Includes one subject who was positive on unscheduled visit (between one-year and two-year visit). (DOCX) [file pone.0268685.s002.docx]

**Supplemental Table A.** Patterns of negative, borderline, positive, and indeterminant quantitative IGRA (qIGRA) results among study subjects (including unscheduled visits).

| **Baseline**  **(*n* = 928)** | **A** | **2 months**  **(*n* = 648)** | **B** | **C** | **D** | **1 year**  **(*n* = 627)** | **E** | **F** | **2 years**  **(*n* = 588)** | **G** | **H** | **3 years**  **(*n* = 499)** | **I** | **J** | **K** |
| --- | --- | --- | --- | --- | --- | --- | --- | --- | --- | --- | --- | --- | --- | --- | --- |
| 761  (Negative) | 3 | 613 | 2 |  |  | 566 | 2 | 1 | 505 | 4 | 1 | 405 | 1 |  |  |
|  |  |  |  |  |  |  |  |  |  |  |  | 13 | 3 |  |  |
|  |  |  |  |  |  |  |  |  |  |  |  | 1 | 1 | 1 | 1 |
|  |  |  |  |  |  |  |  |  |  |  |  | 3 | 1 |  |  |
|  |  |  |  |  |  |  |  |  |  |  |  |  | 1 |  |  |
|  |  |  |  |  |  |  |  |  |  |  |  | 2 | 1 | 1 | 1 |
|  |  |  |  |  |  |  |  |  |  |  |  |  | 1 | 1 | 1 |
|  |  |  |  |  |  |  |  |  |  |  |  | 3 |  | 2 |  |
|  |  |  |  |  |  |  |  |  | 7 | 2 |  | 7 |  |  |  |
|  |  |  |  |  |  |  |  |  | 13 | 4 |  | 6 |  |  |  |
|  |  |  |  |  |  |  |  |  |  | 3 |  | 3 | 1 |  |  |
|  |  |  |  |  |  |  |  |  |  |  |  |  | 1 |  |  |
|  |  |  |  |  |  |  |  |  |  | 1 |  | 1 |  |  |  |
|  |  |  |  |  |  |  |  |  |  | 1 |  | 1 |  |  |  |
|  |  |  |  |  |  |  |  |  |  | 1 |  | 1 |  |  |  |
|  |  |  |  |  |  |  |  |  | 8 | 1 |  | 8 |  |  |  |
|  | 1 |  |  |  |  | 5 |  |  | 4 |  |  | 3 |  |  |  |
|  |  |  |  |  |  |  |  | 1 | 1 |  |  | 1 |  |  |  |
|  |  |  |  |  |  | 10 |  | 3 | 5 | 2 |  | 1 |  |  |  |
|  |  |  |  |  |  |  |  |  |  |  |  | 3 |  | 1 |  |
|  |  |  |  |  |  |  |  |  | 2 |  |  | 2 |  |  |  |
|  |  |  |  |  |  |  |  |  | 1 | 1 |  | 1 |  |  |  |
|  |  |  |  |  |  |  |  |  | 1 |  |  | 1 |  |  |  |
|  |  |  |  |  |  | 9 |  |  | 5 |  |  | 5 |  |  |  |
|  |  |  |  |  |  |  |  |  | 1 |  |  | 1 |  |  |  |
|  |  | 5 | 1 | 1 | 1 | 5 |  |  | 4 |  |  | 3 |  |  |  |
|  |  |  |  |  |  |  |  |  | 1 |  |  | 1 |  |  |  |
|  |  | 15 | 10 | 3 |  | 1 |  |  | 1 | 1 |  | 1 |  |  |  |
|  |  |  |  |  |  | 1 |  |  |  |  |  | 1 |  |  |  |
|  |  |  |  |  |  | 1 |  |  | 1 |  |  | 1 |  |  |  |
|  |  |  |  | 2 |  | 2 |  |  | 2 |  |  | 2 |  |  |  |
|  |  |  |  |  |  | 1 |  |  | 1 |  |  | 1 | 1 |  |  |
|  |  |  |  |  |  | 3 |  |  | 1 | 1 |  |  |  |  |  |
|  |  |  |  |  |  |  |  |  | 1 |  |  |  |  |  |  |
|  |  |  |  |  |  | 1 |  |  | 1 |  |  | 1 |  | 1 |  |
|  |  |  | 4 | 2 |  | 2 |  |  | 1 |  |  | 1 |  | 1 |  |
|  |  |  |  |  |  |  |  |  | 1 | 1 |  | 1 |  |  |  |
|  |  |  |  | 2 |  | 2 |  |  | 2 | 1 |  | 1 |  |  |  |
|  |  |  |  |  |  | 1 |  |  | 1 |  |  | 1 |  |  |  |
|  |  | 3 |  |  |  | 3 |  |  | 2 |  |  | 1 |  |  |  |
|  |  |  |  |  |  |  |  |  | 1 |  |  |  |  |  |  |
| 21  (Borderline) |  |  |  |  |  |  |  |  |  |  |  |  |  |  |  |
|  |  | 4 |  |  |  | 4 |  |  | 3 |  |  | 1 |  |  |  |
|  |  | 1 |  |  |  | 1 |  |  | 1 |  |  | 1 |  |  |  |
|  |  | 3 | 1 | 1 |  | 1 |  |  | 1 |  |  | 1 |  |  |  |
|  |  |  | 1 |  |  | 1 |  |  | 1 |  |  | 1 |  |  |  |
|  |  |  |  |  |  | 1 |  |  | 1 |  |  | 1 |  |  |  |
| 143  (Positive) | 1 |  |  |  |  |  |  |  |  |  |  |  |  |  |  |
|  |  | 1 |  |  |  | 1 |  |  | 1 |  |  | 1 |  |  |  |
|  |  | 2 |  |  |  | 1 |  |  | 1 |  |  | 1 |  |  |  |
|  |  |  |  |  |  | 1 |  |  | 1 | 1 |  |  |  |  |  |
|  |  |  |  |  |  | 2 |  |  | 3 | 3 |  | 1 |  | 1 |  |
|  |  |  |  |  |  |  |  |  |  |  |  | 2 |  |  |  |
| 3  (Indeterminant) | 1 | 1 |  |  |  | 1 |  |  | 1 |  |  | 1 |  |  |  |

IGRA result: negative = green; borderline = orange; positive = red; indeterminant = grey. Alphabetical columns (A-K) refer to additional visits that occurred between scheduled per-protocol visits (baseline, two-month, one year, two year, and three year). Because of repeated unscheduled visits for a subset of subjects, multiple alphabetical columns may appear between scheduled visits. Note: Alphabetical columns A, B, C, D and E in this table do not correspond to alphabetical columns in Table 7.
