# Supplementary material for: Serial T-SPOT.TB responses in Tanzanian adolescents: Transient, persistent and irregular conversions
Source: PLoS One. 2022 Jun 24;17(6):e0268685. doi: 10.1371/journal.pone.0268685 (PMC9231806; doi:10.1371/journal.pone.0268685)
Supplement: S2 Table — Values are reported as: median [range]. Subjects with first positive IGRA at an unscheduled visit (n = 4) are excluded. SFCs for the Nil, ESAT-6, CFP-10, and PHA wells were compared between two-month (n = 15), one year (n = 10), two year (n = 13), and three year (n = 3) positives at the time of initial positivity (green boxes) using the Kruskal-Wallis H-test. Results were as follows: Nil: p = 0.56, ESAT-6: p = 0.89, CFP-10: p = 0.11, PHA: p = 0.01. In pairwise comparison, PHA was significantly greater in year one positives versus two-month positives (p = 0.003). None of the other pairwise comparisons were statistically significant. (DOCX) [file pone.0268685.s003.docx]

**Supplemental Table B.** Spot forming cells (SFCs) among 41 incident IGRA converters at the 5 per-protocol visits.

| **Timing**  **of conversion** | **Baseline** | **Two months** | **One year** | **Two years** | **Three years** |
| --- | --- | --- | --- | --- | --- |
| **Two months**  **(N=15)**  Nil  ESAT-6  CFP-10  PHA | 0 [0-3]  0 [0-5]  1 [0-4]  195 [40-530] | 0 [0-5]  12 [2-133]  10 [0-405]  330 [75-542] | 0 [0-2]  2 [0-32]  3 [0-84]  286.5 [50-863] | 0 [0-6]  4.5 [0-113]  3 [0-110]  309 [132-1000] | 0 [0-2]  1 [0-22]  0.5 [0-27]  395 [75-552] |
| **One year**  **(N=10)**  Nil  ESAT-6  CFP-10  PHA | 0.5 [0-4]  0 [0-7]  0.5 [0-5]  296.5 [22-527] | 0 [0-2]  0 [0-1]  1 [0-1]  285 [43-598] | 0 [0-2]  23 [4-82]  19 [0-41]  475 [367-1000] | 0 [0-0]  4 [0-89]  8 [0-39]  307 [28-1000] | 0 [0-2]  4 [0-65]  7.5 [0-45]  361 [110-715] |
| **Two years**  **(N=13)**  Nil  ESAT-6  CFP-10  PHA | 0 [0-2]  0 [0-2]  0 [0-2]  203 [30-359] | 0 [0-0]  0 [0-3]  0 [0-3]  201 [60-481] | 0 [0-2]  0 [0-1]  0 [0-3]  187 [38-346] | 0 [0-2]  22 [2-277]  11 [0-224]  355 [157-578] | 0 [0-4]  2.5 [0-189]  5 [0-136]  217 [83-540] |
| **Three years**  **(N=3)**  Nil  ESAT-6  CFP-10  PHA | 0 [0-2]  0 [0-1]  1 [0-4]  237 [167-443] | 1 [0-1]  0 [0-0]  0 [0-3]  165 [152-370] | 0 [0-0]  1 [0-1]  0 [0-1]  238 [137-243] | 0 [0-0]  0 [0-1]  0 [0-0]  211 [174-539] | 0 [0-2]  12 [1-118]  74 [31-157]  148 [112-429] |

Values are reported as: median [range]. Subjects with first positive IGRA at an unscheduled visit (*n* = 4) are excluded. SFCs for the Nil, ESAT-6, CFP-10, and PHA wells were compared between two-month (*n* = 15), one year (*n* = 10), two year (*n* = 13), and three year (*n* = 3) positives at the time of initial positivity (green boxes) using the Kruskal-Wallis H-test. Results were as follows: Nil: *p* = 0.56, ESAT-6: *p* = 0.89, CFP-10: *p* = 0.11, PHA: *p* = 0.01. In pairwise comparison, PHA was significantly greater in year one positives versus two-month positives (*p* = 0.003). None of the other pairwise comparisons were statistically significant.
